# Case of Spontaneous Expulsion of the Child

**Published:** 1850-08

**Authors:** 


*Case of Spontaneous Expulsion of the Child.*—The following case, of what was termed by Denman "*spontaneous evolution*," read before the Medico-Chirurgical Society of Aberdeen, by Dr. Robert Dyce, and published in the Monthly Journal of Medical Science, May, 1850, is interesting, not only for its extreme rarity, but also for the many untoward circumstances which accompanied it:

"I was called about midnight on Saturday, 30th December, 1848, by a midwife, to visit Mrs. C——, the wife of a tradesman, living in Castle street. I was informed that labor had commenced in the evening about six hours before—that the presentation remained long high—that the membranes ruptured naturally—that the waters were in great quantity—and that several strong pains had followed after the discharge of the waters, before any part of the child could be felt—a limb was at length reached, which was made out by her to be an arm. When I first saw the patient she had very strong forcing pains; the arm was at the top of the vagina, doubled up so as to present the elbow. A part of the child, nearly equal in bulk, was felt on either side of the presenting limb, viz.: one part near the pubes, and the other near the sacrum, but so high that unless I had passed my whole hand into the vagina, which I did not at the time deem essential, the individual parts could not be made out. It was sufficient for my purpose that the arm presented, and that delivery could not be accomplished without turning the child. In order, therefore, to render the operation easier, by quieting the uterine action, which was very powerful, I gave her, as soon as it could be procured, a teaspoonful of laudanum, determining to operate the moment a lull took place. This, however, never happened, for presently the pains forced the elbow lower, the hand came down into the vagina with hardly any assistance, and was ascertained now to be the right one. At this time the proportionate size or bulk of the two parts of the child became remarkably altered. The arm, shoulder and neck, which formed one part, pressed towards the pubes, and appeared smaller; while the other end of the tumor, which was now distinctly made out to be the back of the child, along with the ribs and spine, which was twisted and bent, now came completely to occupy the hollow of the sacrum. It now became very apparent that nature was to complete the delivery herself, by expelling the child double, or by what is called spontaneous expulsion. At length, after two or three powerful pains, the shoulder was very closely pressed, or jammed rather, against the arch of the pubes, and at length external to the vulva, while the breech pressed out the perineum, and was expelled by a very long and powerfully continued pain, the feet following quickly in its wake, the arm never moving from its position under the pubes. The head soon followed, and the delivery was speedily completed. The child (a girl) gasped once or twice, but could not be recovered.

The size of the abdomen indicated the presence of another child, which, on examination *per vaginam*, was confirmed, as the membranes were reached. I also discovered in the examination a circumstance by no means desirable, viz.: that the integuments of the abdomen, limbs, face, and in short, the whole body, were extensively edematous, for lying, as the patient did, with her back towards me, I had no opportunity of discovering this before.

No pain coming on in half-an-hour, the membranes were ruptured. The waters were in very great quantity. An arm again presented, but along with the head. It was attempted to keep up the limb, so as to let the head descend alone; but the pains were so violent that both head and arm were forced into the pelvis, and expelled together. The breech remained during two or three equally severe pains at the birth, owing to the cord, which was very short, being twisted round one thigh and leg, by which it was tucked up tight upon the infant's abdomen; its removal immediately caused the expulsion of a second girl, alive and strong. In a quarter of an hour pains returned, but no part of the placenta could be felt; and as the uterus felt contracted, small and tolerably defined, while no hemorrhage at first ensued, no interference was resorted to. Very shortly, however, the pains became very severe, along with hemorrhage to some extent so as to lead me to fear that irregular contraction either had taken place or was impending, and that probably one of the placente might be separated. The hand was immediately introduced. One placenta was found loose in the lower part of the uterus, a portion of the other in the contracted part, while by far the largest portion was imprisoned above, in the upper chamber of the uterus. The usual methods were carefully tried to separate the placenta, viz.: by patting it, by grasping it from its edge to its centre, while the uterus was steadied by the hand on the abdomen externally; but no impression was made upon it. It was then attempted to remove it bit by bit; but so firmly was it attached, and to so very large a surface, that I for a moment hesitated what was best to be done—whether to persevere carefully in my present proceeding, or to leave some of the lobules adhering to the uterus. Both methods were attended with danger; but knowing well the great risk there is in separating a strongly and morbidly-adherent placenta, from the difficulty in distinguishing the soft and loose structure of the womb from the mass of the placenta, I decided on the latter method. I therefore kept my fingers close to the placenta and pinched off several of the lobules and left them adhering to the uterus. The womb now contracted regularly, and expelled my hand and the placenta together. Hemorrhage ceased, and the pulse, when I left at two in the morning, was 86. The woman had a most perfect recovery.

